# Testing a Social Network Intervention Using Vlogs to Promote Physical Activity Among Adolescents: A Randomized Controlled Trial

**DOI:** 10.3389/fpsyg.2019.02913

**Published:** 2020-01-10

**Authors:** Thabo J. Van Woudenberg, Kirsten E. Bevelander, William J. Burk, Crystal R. Smit, Laura Buijs, Moniek Buijzen

**Affiliations:** ^1^Behavioural Science Institute, Radboud University, Nijmegen, Netherlands; ^2^Radboud Institute for Health Sciences, Primary and Community Care, Radboud University and Medical Centre, Nijmegen, Netherlands; ^3^Erasmus School of Social and Behavioural Sciences, Erasmus University Rotterdam, Rotterdam, Netherlands

**Keywords:** social network intervention, physical activity, preventive medicine, accelerometer, adolescents, health, vlogs

## Abstract

There is a need to stimulate physical activity among adolescents, but unfortunately, they are hard to reach with traditional mass media interventions. A promising alternative is to carry out social network interventions. In social network interventions, a small group of individuals (*influence agents*) is selected to promote health-related behaviors within their social network. This study investigates whether a social network intervention is more effective to promote physical activity, compared to a mass media intervention and no intervention. Adolescents (*N* = 446; *M*_*age*_ = 11.35, *SD*_*age*_ = 1.34; 47% male) were randomly allocated by classroom (*N* = 26, in 11 schools) to one of three conditions: social network intervention, mass media intervention, or control condition. In the social network intervention, 15% of the participants (based on peer nominations) was approached to become an influence agent, who created vlogs about physical activity that were shown during the intervention. In the mass media intervention, participants were exposed to vlogs made by unfamiliar peers (i.e., vlogs of the social network intervention). The control condition did not receive vlogs about physical activity. All participants received a research smartphone to complete questionnaires and a wrist-worn accelerometer to measure physical activity. The trial was registered *a priori* in the Dutch Trial Registry (NTR6903). There were no differences in objectively measured physical activity between this social network intervention and the control condition in the short-term, but there was an unexpected increase in the control condition compared to the social network intervention in the long-term. No differences between the social network intervention and mass media intervention were observed. The current study does not provide evidence that this social network intervention is effective in increasing physical activity in adolescents. Exploratory analyses suggest that this social network intervention increased the perceived social norm toward physical activity and responses to the vlogs were more positive in the social network intervention than in the mass media intervention. These initial results warrant further research to investigate the role of the social norms and the added benefit of using influence agents for social network interventions.

**Clinical Trial Registration:** https://www.trialregister.nl/, identifier NTR6903.

## Introduction

Physical activity has a positive effect on youth’s physical (Janssen and LeBlanc, 2010) and mental health (Biddle and Asare, 2011), academic performance (Trudeau and Shephard, 2008), and life satisfaction (Brooks et al., 2014). However, 81% of adolescents worldwide do not adhere to the recommended amount of daily physical activity (Guthold et al., 2019). Youth tend to become less active as they grow older, and adolescents today are less physically active than adolescents in previous generations (Boreham and Riddoch, 2001; Tudor-Locke et al., 2011; Kohl et al., 2012). This is problematic, because (un)healthy habits formed in childhood can persevere into adulthood (Boreham and Riddoch, 2001). Therefore, there is a substantial need for effective interventions to promote physical activity among adolescents.

In the past decades, public health agencies and researchers have used mass media intervention campaigns to promote physical activity at the community level (Cavill and Bauman, 2004; Dobbins et al., 2009). Mass media interventions use standardized messages to increase knowledge, influence attitudes and beliefs, and change behavior (Kahn et al., 2002), and are a relatively inexpensive way of reaching a large audience and, therefore, suitable for large scale implementation (Redman et al., 1990). Although there are examples of mass media interventions that have increased adolescents’ physical activity (e.g., Huhman et al., 2005), a systematic review of traditional mass media campaigns concluded that there is insufficient evidence indicating that mass media campaigns are an effective strategy to promote physical activity in this particular population (Kahn et al., 2002). One of the reasons that mass media interventions do not succeed in increasing physical activity is that people, especially youth, are resistant to information from outside sources (Laverack, 2017). In addition, today’s adolescents are less likely to use traditional mass media and more likely to use online social media (Wartella et al., 2016; Valkenburg and Piotrowski, 2017). Nevertheless, a more recent review showed that interventions that incorporated online social media (e.g., online platforms) as part of the intervention were also not able to increase the amount of physical activity in children and adolescents (Hamm et al., 2014).

Potentially, interventions can be more effective when utilizing the impact that adolescents have on each other’s physical activity by having intervention messages that are communicated by the adolescents themselves in these online social networks (Valente, 2012). Studies in graduate students have shown that the number of enrollments in exercise classes increased when participants were assigned to an online platform that incorporated online social networks compared to an online platform that sends weekly promotional media messages (Zhang et al., 2015, 2016). Therefore, incorporating social relationships in the promotion of physical activity seems promising, by harnessing the effects peers have on each other’s health behaviors (Montgomery et al., 2020).

Social network interventions are an emerging approach to counteract the decline in physical activity, by capitalizing on the influence youth has on each other’s behaviors (Valente, 2012). In social network interventions, a small group of individuals, so-called *influence agents*, are identified based on their central position within each social network (Thoits, 2011). The influence agents are asked to either promote or discourage the targeted behavior within their social network (e.g., classroom), by serving as role models, leaders or advocates of the healthy behaviors. Previous work has shown that social network interventions can stimulate healthy behaviors in the short and long term, such as healthy eating (Shaya et al., 2014) and water consumption (Smit et al., 2016), or discourage unhealthy behaviors, such as smoking (Campbell et al., 2008; Starkey et al., 2009) and substance use (Valente et al., 2007).

Only a few recent studies have adopted the social network approach to promote physical activity among adolescents (Bell et al., 2014; Sebire et al., 2016, 2018; Brown et al., 2017; Jong et al., 2018; Owen et al., 2018; van Woudenberg et al., 2018), However, these studies vary in intervention method, target audience, influence agent selection strategy, and training method. For example, different forms of nominations have been used to select the influence agents (Bell et al., 2014; Sebire et al., 2016, 2018; Brown et al., 2017; Jong et al., 2018; van Woudenberg et al., 2018), and two studies focused on girls only (Sebire et al., 2016, 2018; Owen et al., 2018). In most studies, influence agents received intensive face-to-face training sessions to teach the influence agents how they could promote the behavior within their classroom (Bell et al., 2014; Sebire et al., 2016, 2018; Brown et al., 2017; Jong et al., 2018). One study did not use face-to-face training but trained the influence agents on how to promote physical activity within their class via online training on a research smartphone (van Woudenberg et al., 2018). The majority of the studies, apart from the studies by Bell et al. (2014) and van Woudenberg et al. (2018), successfully increased physical activity in the target group.

However, all previous social network intervention studies on physical activity have used designs in which the effectiveness of the intervention was compared to a control condition that did not receive an intervention. Therefore, these studies cannot distinguish whether the social network interventions were effective because of the exposure to the general message of physical activity promotion compared to a message specifically delivered by influence agents. No previous study compared a social network intervention to a similar intervention without a social influence component (e.g., mass media intervention) to determine the additional benefit of using the social network intervention approach. Therefore, the aim of the current study is to investigate whether a social network intervention is more effective in promoting physical activity than a mass media intervention or no intervention.

In the current study, influence agents will create video blogs (‘*vlogs*’) that will promote physical activity in order to give the influence agents a platform to model and communicate about physical activity behavior (Obrusnikova and Rattigan, 2016), which are known mechanisms to increase physical activity in adolescents (Salvy et al., 2012). Previous studies in adults have shown that the content in tailored videos about physical activity is more accepted and people spend more time on the intervention platform when videos are used in the intervention (Soetens et al., 2014; Vandelanotte et al., 2015). Vlogs are a specific form of short user-generated videos that are available online, for example on YouTube (Gao et al., 2010). Studies on dietary intake in children have shown that when participants were exposed to vlogs in which unhealthy snacks were portrayed, more unhealthy snacks were consumed compared to when participants were exposed to vlogs about non-food products (Coates et al., 2019). Using vlogs as intervention messages connects seamlessly to the purposes of this study, not only because watching vlogs online has become immensely popular among adolescents (Snelson, 2015), but also because it allows for testing the social network intervention principles in a unique and unprecedented way in which the social network intervention condition is exposed to the exact same intervention messages as the mass media intervention. Specifically, to test whether the social network intervention is more effective in increasing physical activity than a mass media intervention or no intervention, participants will be exposed to vlogs created by influence agents within their class (social network intervention), or unfamiliar peers (mass media intervention), or will not be exposed to vlogs about physical activity. The hypotheses are that (1) participants in the social network intervention condition will increase more in physical activity than participants in the mass media intervention condition and (2) participants in the social network intervention condition will increase more in physical activity than participants in the control condition.

Moreover, because no previous studies have investigated the underlying mechanisms of social network interventions, this study will take a first step by exploring secondary outcomes of the intervention. Based on the theory of planned behavior (Ajzen, 1991) and the self-determination theory (Ryan and Deci, 2017), four important secondary outcomes of the intervention are defined: social norms on physical activity, enjoyment of physical activity, self-efficacy of physical activity and motivation to be physically active. Likewise, because of the novelty of using vlogs as intervention messages, there is no precedence in research on how adolescents respond to these types of intervention messages. Therefore, the current study will explore the responses to the vlogs (i.e., exposure to the vlogs, linking of the vlogs and perceived closeness to the vloggers) in the social network intervention and the mass media intervention.

## Materials and Methods

### Design

**FIGURE 1:**
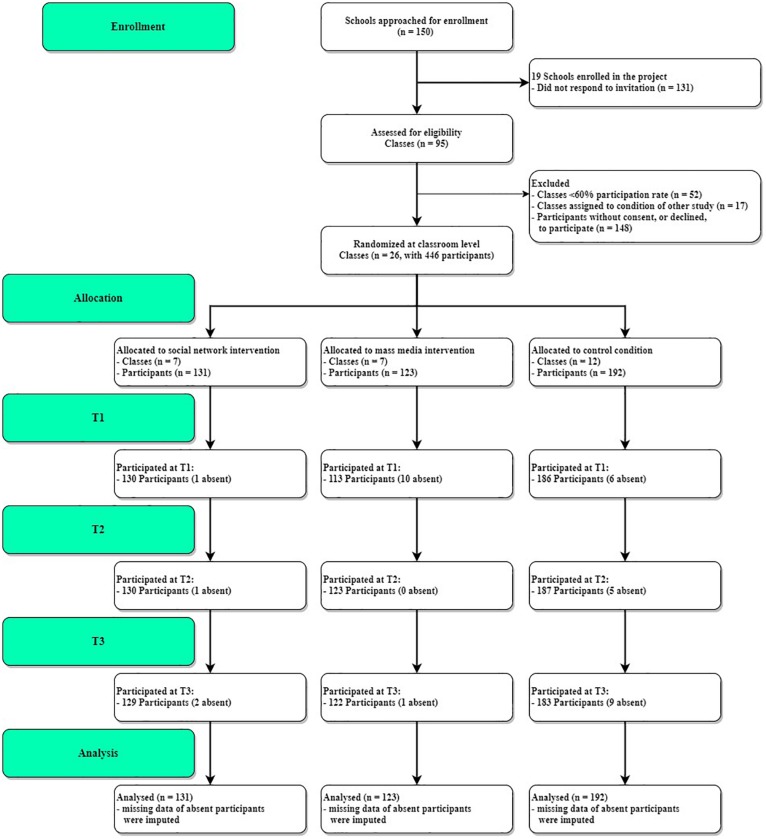
CONSORT flow diagram of the number of participants per condition at the three measurements.

This study used a clustered randomized control trial design with three groups. *A priori*, the study was registered in the Dutch Trial Registry (NTR): TR6903 and procedures were approved by the Ethics Committee of the Radboud University (ECSW2014-100614-222). The required sample size was based on the previous study by Sebire et al. (2018) that found an effect of the social network intervention in a study with 272 adolescents in the intervention and the control condition. This number was multiplied by 1.5 to add the third condition, which resulted in a minimum number of 408 participants (approximately 21 classrooms of 20 participants per class). To account for non-response in the active consent procedure and associated strict exclusion criteria for classes, we approached more than 21 classrooms for participation in the project, see Figure 1.

### Participants and Procedure

The study is part of a two-phase project called the *MyMovez* project. In the first phase, 21 primary and secondary schools were enrolled (for more information see Bevelander et al., 2018). All participating schools were invited for the second phase (intervention phase), and new schools were approached to complement the sample, of which six new schools signed up for participation. All schools sent out information letters and consent forms to the parents or legal guardians of students in the targeted classrooms. To obtain representative samples within each classroom, only classes could participate in which at least 60% of students had active parental consent (Marks et al., 2015). As a result, 43 classes were enrolled in the sample. In total, active parental consent (in accordance with the Declaration of Helsinki) was obtained for 745 students.

In the *MyMovez* project, two separate intervention studies were conducted with a shared control group (i.e., one intervention focused on promoting water drinking and the current intervention on the promotion of physical activity). A total of 19 schools (43 classes) were assigned to one of the five conditions (two water-drinking conditions, two physical activity conditions, and a control condition). Because only four secondary schools participated in the project, the two smallest secondary schools were combined, and the secondary schools were randomly assigned to one of the physical activity conditions or control condition. Thereafter, the primary schools were stratified based on size and randomly assigned over all five conditions. The control condition received relatively more classes because that condition was also part of the other intervention study that only focused on primary schools.

This study included three conditions (two physical activity intervention conditions and the control condition). The sample consisted of 446 participants (47% male) ranging from 9 to 16 years old (*M* = 11.35 years, *SD* = 1.34), from 26 classes in 11 different schools. Seven classes (*n* = 131) were assigned to the social network intervention condition, seven classes (*n* = 123) were assigned to the mass media intervention condition, and 12 classes (*n* = 192) to the control condition. Before receiving the materials, all participants provided informed assent.

The study was divided into three measurement periods: the baseline measurement (February-March 2018; T1), the intervention measurement (April–May 2018; T2), and the follow-up measurement (May–June 2018; T3). In all three measurement periods, the participants used the *MyMovez Wearable Lab* for seven consecutive days: A smartphone with a tailor-made research application and a wrist-worn accelerometer. At the beginning of the study (T1), all participants received instructions about the usage of the *Wearable Lab*. The smartphone was used as an assessment tool, an online social platform for participants within the class, and a means of communication between the researchers and the participants. On the smartphone, participants received daily questionnaires at random moments between 7:00 AM and 7:30 PM, excluding hours that participants were in school. In addition, participants nominated peers on the sociometric questions in this measurement period. The accelerometer measured the amount (i.e., number of steps) and intensity of physical activity per day.

During the intervention week (T2), participants received the *Wearable Lab* to measure the physical activity and fill out short questionnaires again. Also each morning in the intervention week, a new vlog about physical activity was unlocked under the video tile on the research smartphone and participants were told that each day new vlogs became available in the research app. They were able to watch the vlogs as many times as they wanted and share them with peers in their class. To assess the long term effects of peer modeling as portrayed in the vlogs (Hardman et al., 2011), participants received the *Wearable Lab* for the follow-up measure (T3), 5 weeks after the intervention. Again, the daily physical activity and the other variables were measured. The participants had the *Wearable Lab* only during T1, T2, and T3, which means that the amount of physical activity and other variables were not measured between the measurement periods and that the participants could not watch the vlogs before or after the intervention measurement (T2).

### Measures

#### Physical Activity

The wearable accelerometer (Fitbit Flex) measured the number of steps per day. The accelerometer was blinded so that the participants could not see the number of steps that they accumulated per day. Based on previous work (van Woudenberg et al., 2018), incomplete days (<1,000 steps or <1,440 min per 24 h) of measurement were excluded from the analysis. Reasons for incomplete days were the first and last day of the measurement week, the device was not worn, or the battery was empty. In total, 76.5% of all possible data points were observed in the daily physical activity data. When participants had less than 3 days of observed data but at least 1 day of data, single multilevel (predictive mean matching) imputation (Van Buuren, 2011) was used to generate imputed physical activity data (based on 500 iterations). The data points were imputed based on other physical activity data of the participant, class, school, day of the week, gender, age, BMI, weather conditions of that day, and psychosocial measures of the participant (i.e., athletic competence, perceived social norms; enjoyment; self-efficacy and motivation). On average, participants accumulated 9849.69 (*SD* = 5838.63) steps per day and were moderate-to-vigorously active for 55.13 (*SD* = 50.89) minutes per day (as defined by the Fitbit). The imputed values did not differ from the observed values of physical activity, *t*(9548) = 1.62, *p* = 0.11.

#### Sociometric Nominations

Participants nominated peers on five sociometric questions. Three questions were based on previous studies (Campbell et al., 2008; Starkey et al., 2009) that used peer nominations to identify influence agents (i.e., “Whom do you ask for advice?”; “Who in your classroom are leaders, or take the lead often?”; “Who do you want to be like?”). The remaining two questions (Salvy et al., 2012) were included to identify adolescents interact (i.e., “With whom do you hang out during the breaks?”) and communicate about physical activity with each other (i.e., “With whom do you talk about physical activity?”). Participants nominated peers within the same grade at school. In addition, participants could search for names in the provided search field and were required to nominate at least one other peer (self-nominations were impossible). This study focused on interventions within classrooms, therefore only nominations with the same classroom were included. Based on these five sociometric nominations, one aggregated social network per classroom was created.

In general, social network interventions use the most often nominated participants as selection criteria for influence agents (i.e., those with the highest *in-degrees*). However, in school settings, these participants are also the most popular adolescents and are clustered together in the network, which limits the reach of the influence agents. Also, popular peers may be reluctant to change their behavior or perform the role of an influence agent in order to retain their social status (Valente, 1995). Therefore, the group of participants that collectively had the highest closeness centrality was selected as influence agents (van Woudenberg et al., 2018). Closeness centrality is the average distance between the participant and all other peers in a network. More specifically, closeness central individuals have the shortest paths to all other peers, making them the most strategic influence agents to disseminate the health message in a social network in the least amount of time (Borgatti, 2005).

**FIGURE 2:**
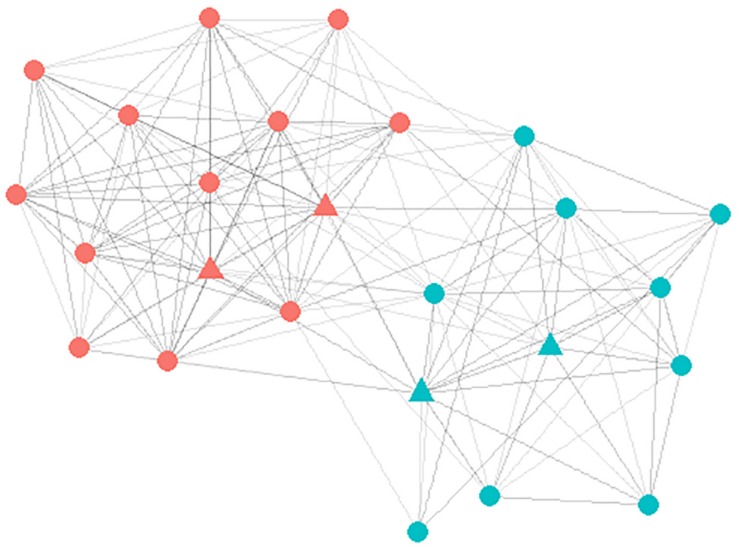
Visual representation of the social network of one of the social network intervention classes. The dots represent the participant (nodes) and the lines between the dots represent the connection (edges). The blue nodes are males and the red nodes are female. The targeted influence agents are marked by a triangle.

The KeyPlayer package (version 1.0.3; An and Liu, 2016) in R (R Core Team, 2013) was used to determine a specified number of influence agents that collectively represented the group of participants that are the most closely related to all other peers, adjusting for overlapping nominations within each classroom network (An and Liu, 2016). Based on previous studies (Valente and Pumpuang, 2007; Rogers, 2010), 15% of males and 15% of females were identified as influence agents in each classroom (Araujo et al., 2018). All 22 of the participants who were approached accepted the role of influence agent. In one school, three participating classes shared one room in the building (as part of the teaching philosophy). Therefore, students in these three classes were combined into one large network for the influence agent selection procedure. This resulted in four intervention classes with four influence agents, and the last intervention classroom with six influence agents. Figure 2 represents the social network of one of the participating classrooms. The dots represent the participants in the class and the influence agents are represented by the triangles. The blue nodes are males and the pink nodes are females.

#### Social Norms

Perceived descriptive norms of classmates was measured by a single item: ‘How many days per week are your classmates physically active for more than an hour per day?’ Perceived injunctive norms of classmates was measured by a single item: ‘How many days per week do your classmates think that you should be physically active for more than an hour per day?’ For both questions, participants could answer between 0 and 7 days per week (Pedersen et al., 2015).

#### Enjoyment

Enjoyment was measured by a Visual Analog Scale (VAS). Participants were asked to indicate how much they enjoyed sports and physical activity, and could answer by placing their finger on a slider ranging from 0 (‘not at all fun’) to 100 (‘very much fun’).

#### Self-Efficacy

Self-efficacy was measured by two items (van der Horst et al., 2007): ‘Do you think you are able to be more physically active?’ and ‘Does being more physically active seem difficult to you?’. Participants could answer on a 6-point Likert scale ranging from ‘No, definitely not’ to ‘Yes, definitely.’ The two items (*r* = 0.62, *p* < 0.001) were averaged into one variable.

#### Motivation

Motivation was measured by 12 items, in four subdomains as described in the self-determination theory (Ryan and Deci, 2017): extrinsic, introjected regulation, identified regulation, and intrinsic. Participants read statements describing the different types of motivation (e.g., ‘I am physically active because I think this is important’) and could answer on a 6-point Likert scale ranging from ‘No, not at all’ to ‘Yes, definitely’. The extracted means at each time point are reported in Table 1.

**TABLE 1:** Overview of measures.

		**Items**	**α**	**Range**	***M_T__1_***	***SD_T__1_***	***M_T__2_***	***SD_T__2_***	***M_T__3_***	***SD_T__3_***
Steps per day		–	–	[1,000 – 44,560]	9181	(5038)	10910	(6506)	9479	(5781)
Social norms	Descriptive	1	–	[0–7]	3.84	(1.76)	3.77	(1.81)	3.74	(1.92)
	Injunctive	1	–	[0–7]	3.47	(2.20)	3.65	(2.21)	3.62	(2.15)
Enjoyment		1	–	[1–6]	5.13	(1.05)	5.18	(0.94)	5.13	(1.02)
Self-efficacy		2	–	[1–6]	4.93	(1.09)	4.88	(1.18)	4.52	(1.34)
Motivation	Extrinsic	3	0.68	[1–6]	1.48	(0.94)	1.71	(1.24)	1.75	(1.27)
	Introjected	3	0.78	[1–6]	2.03	(1.23)	2.1	(1.35)	2.05	(1.40)
	Identified	3	0.85	[1–6]	4.91	(1.12)	4.93	(1.13)	4.72	(1.31)
	Intrinsic	3	0.59	[1–6]	5.37	(0.90)	5.27	(0.94)	5.16	(1.07)

**N* = 446.*

#### Vlogs Exposure

Vlog exposure was measured by how many times, and seconds a participant watched the vlogs. The first vlog (introduction vlog) was excluded because it did not promote physical activity. The five remaining vlogs had an average length of 50.36 s (*SD* = 22.00). On average, the vlogs were watched 1.67 times (*SD* = 3.06) per participant per day, with an average viewing time of 60.22 s per participant per day (*SD* = 116.64).

#### Liking of the Vlogs

The liking of the vlogs was measured at the end of each day on which the vlog became available by a VAS on which the participants indicated how much they liked the vlog, ranging from ‘not fun’ (0) to ‘very much fun’ (100). On average, the vlogs were evaluated slightly positive (*M* = 58.14, *SD* = 34.31).

#### Perceived Closeness With the vloggers

The perceived closeness with the vloggers was assessed on the last day of T2 (when participants had received all vlogs) and was measured by using the *Inclusion of Other in the Self-*scale (Aron et al., 1992). Participants indicated which image best represented the overlap between themselves and the vloggers ranging from an image with two circles that do not overlap (1) to an image with two almost completely overlapping circles (7). Participants reported on average a moderate degree of closeness to the vloggers (*M* = 4.09, *SD* = 1.90).

#### Covariates

Sex and age were included to correct for possible confounding effects because males tend to be more active than females, and younger adolescents tend to be more active than older adolescents (Sallis et al., 2000; Sherar et al., 2007). In addition, participants’ weight and height were measured individually by a trained research assistant according to standard procedures (with clothes, but without shoes) at T1. Based on the weight and height, the Body Mass Index was calculated, *M* = 18.05, *SD* = 3.15 (5.2% overweight, 0.7% obese). BMI was used to calculate a standardized measure of BMI, based on the age and sex of the participant, which accounts for variations in the growth curves of youth (Schönbeck et al., 2011). The type of school (primary and secondary) was also added as a covariate to control for structural differences between the two types of schools. Adolescents are less active in the weekend compared to weekdays (Lee et al., 2016), so a variable distinguishing weekdays from weekends was added as a covariate.

Also, the perceived athletic competence was added as a covariate, measured at T1 and T3 by the *physical* subscale of the *Children’s Perceived Competence Scale* (Nagai et al., 2015). The subscale consisted of 10 items measuring the perceived level of in physical activity (e.g., “Are you good at sports?” or “Do you have confidence in doing new sports for the first time?”) measured on a 6-point Likert scale (α = 0.78) ranging from “no, definitely not” to “yes, definitely,” Cronbach’s α = 0.78, *M* = 4.34, *SD* = 0.87. Lastly, the weather conditions (mean temperature, hours of sunshine, hours of precipitation, and humidity) as provided by the Royal Dutch Meteorological Institute (2019) were used as covariates to control for the effect of the weather on physical activity. Supporting data and syntax can be found in Supplementary Data Sheet S1.

### Conditions

#### Social Network Intervention

The influence agents were approached on the last day of T1 and were invited to create vlogs about physical activity. The influence agents who accepted their role (100%) watched six short video instructions on a laptop screen, presented by a famous Dutch vlogger. In the instruction, they learned how to write scenarios, how to present and how to film the content of the vlogs, but they did not receive training in how they can influence the physical activity of peers. After watching the instruction videos, the influence agents received an example script and a range of topics that they could use as inspiration for each vlog (“YourMovez,” “Motivate,” “Challenge Time,” “Boys vs. Girls,” and “Don’t be a couch potato”). These topics were based on different social influence components (i.e., social norm, enjoyment, self-efficacy, and motivation; Ajzen, 1991; Salvy et al., 2008, 2012; Ryan and Deci, 2017). The topics targeted the increase in social norms by showing how physically active the influence agents are, increasing enjoyment by showing fun ways to be active, increasing the ability to be physically active by providing new types activities and increasing the motivation to be physically active by providing challenges. The influence agents were free to come up with their own topics, which none of the group did. For each topic, four *cheat cards* were given to the groups of influence agents when they needed inspiration for the content of the vlogs. These cards suggested in one or two sentences the content that could be filmed for this specific topic. Most of the group of influence agents requested to look at one or multiple cheat cards.

The influence agents filmed the content for the first vlog with the help of the researcher. Afterward, the influence agents could ask questions and schedule meetings with the other influence agents to film the remaining vlogs. In addition, each group of influence agents also received a sheet with crude ideas for the remaining vlogs. During the process, it was stressed by the researcher that the assignment should remain a secret to the rest of their classroom until after the vlogs had been shown. All influence agents promised to keep this secret from the rest of the class.

On the first day of the intervention period, all participants were instructed in the classroom that a select group of influence agents had created vlogs about physical activity and that the participants were able to see the vlogs on the provided smartphone. Each morning (at 7:00 AM) a new vlog became available under the ‘vlog tile’ in the *MyMovez* app. Participants could watch the vlogs as often as they wanted to, give *likes* on the vlogs and send the vlogs to classmates via the social platform of the research phone. On average, the participants in the social network intervention conditions watched the vlogs 15.69 times (*SD* = 20.60) across the entire week, with a total viewing time of 515 s (*SD* = 641) per participant, but 22 participants in the social network intervention conditions (8.6%) did not watch any of the vlogs.

#### Mass Media Intervention

Similar to the social network intervention, all participants were instructed in the classroom that vlogs about physical activity would become available daily on the provided smartphone. The vlogs from the social network intervention condition were used and matched in terms of school type (primary and secondary). As a result, the vloggers were unfamiliar peers, resembling a mass media campaign that adolescents are exposed to on the internet. Each vlog was presented once in the social network intervention condition and once in the mass media intervention condition. On average, participants in the mass media intervention condition watched the vlogs 7.21 times (*SD* = 14.60), with an average viewing time of 218 s (*SD* = 444), 40 participants did not watch any of the vlogs.

#### Control Condition

The control condition was not exposed to vlogs about physical activity. In the research application, other short videos were available (e.g., animal bloopers, animations, pranks, or pop-culture videos), which were also available for participants in the other conditions.

## Results

### Strategy of Analysis

The data were handled and analyzed in R (R Core Team, 2013). To control for the hierarchical structure of the data, a mixed-effects model approach was used (Singer and Willett, 2003; Barr et al., 2013). Mixed-effects models were performed with the lme4 package (Bates, 2010). Statistical significance (*p*-values) were determined using the Satterthwaite approximation (Luke, 2017).

The preparatory analyses were used to identify the most appropriate random effects structure for the data. More specifically, variance in physical activity explained by each level (i.e., school type, school, class, participant, weekend, date) was compared separately to an intercept-only model. Based on the ICC, each level was added in a stepwise approach when the model fit improved significantly as indicated by a statistically significant chi-square difference test. After the random effects structure was identified, a mixed-effects model with the condition (social network intervention, mass media intervention, and control) included as a fixed effect was performed on the physical activity data of T1 to test whether the randomization was successful.

The primary analysis used a mixed-effect model to test differences between conditions in physical activity over time. More specifically, condition, time, the interaction between condition and time, as well as several covariates (i.e., sex, age, BMI, athletic competence, and weather conditions) were included as fixed effects in the model. Because condition and time were both categorical variables with three factor levels, two planned contrasts were used to test differences between conditions and between time periods. For condition, the first contrast compared the social network intervention to the control condition, and the second contrast compared the social network intervention to the mass media intervention. For time, the first contrast compared T1 with T2 to assess short-term effects, and the second contrast compared T1 with T3 to assess long-term effects. As sensitivity analyses, the same analysis was repeated twice, once without the imputed data and once without the data of the influence agents.

Additional mixed effect models tested differences between the three conditions on several secondary outcomes: perceived social norms, physical activity enjoyment, self-efficacy, and motivation. For each outcome, an identical model specification was used as in the main analyses, with the only adjustment being the physical activity variable was substituted for the respective outcome variable.

The last set of analyses were limited to participants in the two conditions that were exposed to the physical activity vlogs (i.e., the control conditions was excluded). The analyses investigated whether the amount of exposure to the vlogs, liking of the vlogs and the perceived closeness to the vloggers was higher in the social network intervention condition compared to the mass media intervention condition, by using *t*-tests.

### Preparatory Analyses

#### Clustering of Data

Due to the complex design of the study, the amount of variance in physical activity that could be attributed to differences between the levels of data (i.e., school type, school, class, participant, weekend, date) was initially assessed. Per level, a separate random-intercept model was performed and compared to an intercept-only model (no random intercept) and the ICC per level was calculated. The variance in physical activity could mostly be attributed to differences between participants (ICC = 0.19), and a likelihood ratio test indicated that a random intercept per participant improved the model fit, χ^2^(1) = 551.23, *p* < 0.001. Second, the random intercept of the date (ICC = 0.08) was added, which improved the model fit again, χ^2^(1) = 325.98, *p* < 0.001. Lastly, the random intercept of classroom (ICC = 0.03) was added, which also improved the model fit, χ^2^(1) = 6.72, *p* = 0.009. To conclude, subsequent models included random intercepts per class, participant and date.

#### Randomization Check

To test whether there were differences in physical activity at T1 between the conditions, a mixed-effects model showed that the social network intervention condition did not differ in physical activity from the mass media intervention, *b* = 844.71, *SE* = 783.12, *p* = 0.533, or the control condition, *b* = 81.88, *SE* = 557.23, *p* = 0.988. Also, the mass media intervention condition did not differ from the control condition, *b* = 762.83, *SE* = 721.24, *p* = 0.546. Thus, this analysis indicated that the randomization was successful.

### Main Analyses

**FIGURE 3:**
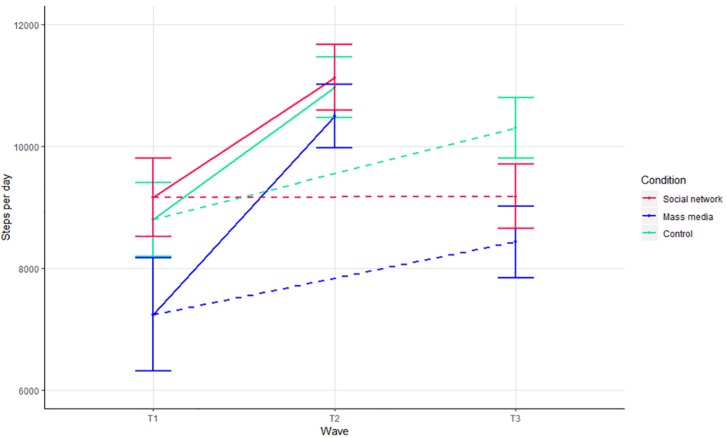
Mean steps per measurement and condition. Estimated marginal mean steps per day for the three conditions at the three time-points, controlling for the clustering in data and all covariates. Error bars represent standard errors.

Table 2 presents the unstandardized model estimates for the primary analysis. Only one of the four interaction effects testing differences between conditions over time emerged as statistically significant. Specifically, the interaction (labeled *Long term ^∗^ control vs. SNI*) indicated that the increase in physical activity from T1 to T3 was greater for participants in the control condition compared to those in the social network intervention. Therefore, there is no evidence that this social network intervention was more effective in increasing physical activity in adolescents compared to the mass media intervention or no intervention. A main effect for the short-term contrast also emerged as statistically significant, indicating that participants in all three conditions increased in physical activity from T1 to T2. Figure 3 presents the estimated means and standard errors for physical activity separately for the three conditions and three time-points. The short-term contrasts comparing differences between the conditions from T1 and T2 are depicted by the solid lines in Figure 3. The long-term contrasts comparing the differences between the conditions from T1 and T3 are depicted by the dotted lines in Figure 3.

**TABLE 2:** Estimates of the mixed effects model.

		**s^2^**	***B***	***SE***	***DF***	***t*-value**	***p***
Random	Class	0.003					
	Child	0.18					
	Date	0.05					
Fixed	(Intercept)		9, 525.63	235.70	49.49	40.41	< 0.001
	Condition: MMI vs. SNI		–200.23	406.91	20.48	–0.49	0.628
	Condition: control vs. SNI		1, 099.32	518.85	33.81	2.12	0.042
	Short term		2, 460.11	866.12	64.75	2.84	0.006
	Long-term		904.33	870.56	64.71	1.04	0.303
	Sex: male vs. female		847.19	271.06	431.44	3.13	0.002
	Age (c)		–472.17	147.49	100.86	–3.20	0.002
	BMI (z)		–180.07	118.71	431.27	–1.52	0.130
	Mean temperature (c)		–102.67	56.23	64.99	–1.83	0.072
	Hours of sunshine (c)		154.38	56.30	62.04	2.74	0.008
	Hours of precipitation (c)		–152.56	137.28	60.70	–1.11	0.271
	Humidity (c)		65.17	19.98	58.42	3.26	0.002
	Athletic competence (c)		787.73	160.09	434.47	4.92	< 0.001
	Weekend: week vs. weekend		−1, 081.61	362.17	57.97	–2.99	0.004
	Type: primary vs. secondary		61.18	490.88	48.65	0.12	0.901
	Short term ^∗^ control vs. SNI		–197.63	482.24	2068.60	–0.41	0.682
	Short term ^∗^ MMI vs. SNI		−1, 287.78	860.40	100.96	–1.50	0.138
	Long-term ^∗^ control vs. SNI		−1, 484.66	484.38	1929.41	–3.07	0.002
	Long-term ^∗^ MMI vs. SNI		−1, 172.62	925.66	124.55	–1.27	0.208

**N*_*participants*_ = 446, *N*_*observations*_ = 5388. Marginal *R*^2^ = 0.08, Conditional *R*^2^ = 0.29. MMI, mass media intervention; SNI, social network intervention; (c), centered; (z), standardized.*

As sensitivity analyses, the same models were performed on a subsample of participants with complete information (i.e., excluding the imputed values) and on a subsample that excluded the influence agents. Both of these models revealed an identical pattern of short and long-term interaction effects. Likewise, no significant interaction effects were observed with a planned contrast that compared the mass media intervention and the control condition, meaning that there is no evidence that the mass media intervention outperformed the control condition.

### Exploratory Analyses

#### Secondary Outcomes

**FIGURE 4:**
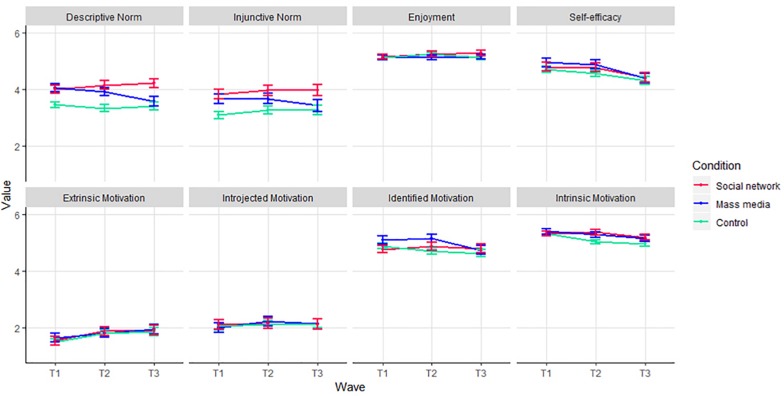
Secondary outcomes per measurement and condition. Estimated marginal means for the secondary outcome variables for the three conditions at the three time-points. Descriptive and injunctive norm and enjoyment variables were scaled to a score in the range between 1 and 6, similar to the range of the other variables. Error bars represent standard errors.

The same mixed-effect models were performed to explore differences between the conditions on the secondary outcomes (perceived social norms, physical activity enjoyment, self-efficacy, and motivation). For each variable, a separate model was performed with the secondary outcome as the dependent variable. Figure 4 presents the differences between conditions over time. Only one of the thirty-two interaction effects emerged as statistically significant. For descriptive norms, participants in the social network intervention differed from those in the mass media intervention from T1 to T3, *b* = 0.83, *SE* = 0.32, *p* = 0.009. The estimated means presented in Figure 4 suggest that descriptive norms involving physical activity increased in the social network intervention and decreased in the mass media intervention.

#### Responses to the Vlogs

The last set of analyses explored whether exposure, liking of the vlogs, and the perceived closeness to the vloggers differed for participants in the social network and mass media interventions (*n* = 254).

The first analysis investigated whether participants in the social network intervention were exposed more often to the vlogs than participants in the mass media intervention condition. Participants in the social network intervention condition watched the vlogs more often during the intervention week (*M* = 15.69, *SD* = 20.60) than participants in the mass media intervention condition (*M* = 7.21, *SD* = 14.60), *t*(1000) = 7.67, *p* < 0.001.

The second analysis investigated whether participants in the social network intervention liked the vlogs better than participants in the mass media intervention. On average, the vlogs were rated significantly more positively in the social network intervention (*M* = 69.09. *SD* = 30.42) compared to the mass media intervention (*M* = 40.20. *SD* = 32.72), *t*(306.73) = 8.88, *p* < 0.001.

The third analysis investigated whether participants in the social network intervention perceived the vloggers as closer than participants in the mass media intervention. On average, the perceived closeness to the vloggers was higher in the social network intervention (*M* = 4.68. *SD* = 1.61) than in the mass media intervention (*M* = 3.46. *SD* = 1.97), *t*(739.75) = 9.54, *p* < 0.001. So overall, responses to the vlogs were more positive in the social network intervention than in the mass media intervention.

## Discussion

This study was the first to investigate the additional benefit of implementing a social network approach to promote physical activity by comparing a social network intervention to a mass media intervention and a control condition. In addition, this study was the first social network intervention using vlogs as intervention messages. While all adolescents increased their physical activity in the short term (from T1 to T2), those in the social network intervention condition increased *less* in the long term compared to the control condition (from T1 to T3). No differences between this social network intervention and a mass media intervention were observed, in either the short or the long term. Therefore, the study does not provide evidence that this social network intervention is more effective in increasing physical activity in adolescents than the mass media intervention or no intervention.

Our findings are not in line with the majority of social network interventions on physical activity (Sebire et al., 2016, 2018; Brown et al., 2017; Jong et al., 2018; Owen et al., 2018). A possible explanation is that in those effective interventions, influence agents received extensive training on how they could promote physical activity. One of the previous studies that did not find an effect of the social network intervention only used an online training of influence agents (van Woudenberg et al., 2018) and discussed that less personal contact might have resulted in a lack of commitment and team effort within the group of influence agents. In the current study, the influence agents did have face-to-face interaction with the researcher and were supported in making the vlogs, but did not receive formal training on how they could promote physical activity within the classroom. Possibly, a key factor to the effectiveness of social network interventions is face-to-face meetings in combination with a training for the influence agents. This would explain why no differences between the two intervention conditions were observed, because neither intervention condition received a formal training on how to promote physical activity. Future studies should test this in a design in which a face-to-face training and an online training is compared to a condition without any training of influence agents.

It was surprising that the physical activity of adolescents in the control condition also increased over time, and even more so than the two intervention conditions. Despite our efforts to find a potential explanation of why the control condition increased the most in physical activity, we could not find a reasonable explanation. For example, we controlled for possible confounding effects of school type (primary and secondary school), differences between the timing of the measurements by including a random intercept per date and specifying the effects of the weather on physical activity as covariates in the model. Likewise, we ruled out an effect of the timing of the measurements because they were evenly divided between the different conditions over time. Lastly, we deemed experimenter and novelty effects unlikely because all three conditions contained both classes that were participating in the first phase of the project and classes that were new to the project. In addition, these two groups of classes showed similar patterns of physical activity. Looking only at the classes that participated in the first phase, we observed only an increase in T2, which for them was the sixth time that they received the wearable lab. Apart from the control condition, the patterns in physical activity of both interventions correspond to a pattern that might be expected for an intervention that is effective in the short term. That is, there is an increase in physical activity as a result of the intervention from T1 to T2, but there is a decrease in physical activity from T2 to T3 because the effect of the intervention dissipates over time. Replication of this study is warranted to corroborate this idea or confirm that in order for a long term effect of the intervention, several boosters or reminders are required to maintain the short term increase in physical activity.

Exploration of secondary outcomes indicated that this social network intervention increased the descriptive norm of physical activity while the mass media intervention decreased the descriptive norm. No other differences were observed in the secondary outcomes between the three conditions. The general lack of statistically significant differences between conditions in these analyses provides some indication of why no differences between the conditions were observed for physical activity. For example, participants reported high levels of enjoyment, self-efficacy, and motivation to be physically active in all conditions, indicating a possible ceiling effect. Possibly, the participants in both interventions already enjoyed physical activity, were able to be physically active, and were sufficiently intrinsically motivated and, therefore, the interventions could not increase these dimensions. However, the exploratory analyses also suggested that descriptive norms increased after exposure to the vlogs in the social network intervention condition, whereas the descriptive norms in the mass media intervention condition decreased. Potentially, when adolescents watch their classmates being physically active, their perceived norm for physical activity increases because they observe that peers from their social group are more physically active than initially perceived. In contrast, when adolescents watch vlogs about physical activity made by unfamiliar peers (who are part of another social group), they perceive another social group as more physically active, and their own social group as less physically active. Future studies should investigate the role of perceived social norms (both descriptive and injunctive) in social network interventions and test whether changes in perceived social norms operate as an underlying mechanism of social network interventions.

The exploratory analyses on the responses to the vlog indicated that in the social network intervention condition, the vlogs were watched more often, rated higher, and the vloggers were perceived as closer to the participants. This is in line with the expectations, because in this social network intervention, the vloggers were classmates of the participants, whereas in the mass media intervention, the vloggers were unfamiliar peers. Potentially, having adolescents within each classroom that create the intervention messages will ensure that participants will be more often exposed to the intervention message and enjoy the intervention more. Nevertheless, this difference did not affect the effectiveness of the interventions.

### Strengths and Limitations

The most important strengths of this randomized controlled trial are that both the design and the intervention messages enabled us to compare a social network intervention to a mass media intervention with identical intervention messages. Additionally, advanced statistical methods have been used to impute missing values, account for the clustering of data within the different levels, and systematically investigate the research questions and exploratory analyses. However, a number of limitations should be discussed when interpreting the results.

First, compared to other social network interventions on physical activity (Bell et al., 2014; Sebire et al., 2016, 2018; Brown et al., 2017; Jong et al., 2018; Owen et al., 2018), the measurement periods were rather short (i.e., 5 days of physical activity data), because of the limited battery life of the accelerometers. Longer measurement periods could ensure a more complete measure of physical activity. In addition, it was not feasible to have more than six vlogs per group of influence agents; otherwise, the burden for the influence agents would be too high. As a result, we were limited in the length of the intervention. It is plausible that psychosocial constructs (e.g., social norms) diffuse through networks faster than actual behavior change and a longer intervention period might have had a significant effect on physical activity of the adolescents. On the contrary, a shorter intervention period increased external validity. In practice, schools have only a limited time to spend on projects outside of their curriculum and more days of data gathering will lead to an increased burden for the participating adolescents, potentially causing additional attrition.

Second, because active consent was required for participation, a sampling bias might have occurred. During the consent procedure, we were under the impression that parents of healthier participants (in terms of BMI) were more likely to provide consent, and that less healthy adolescents were less likely to participate. As a result, the sample could have been biased toward relatively healthy classrooms. Likewise, in each classroom, the relatively healthy adolescents of the class could have participated. Unfortunately, we do not have data on those adolescents that did not participate. Therefore, we could not test whether unhealthy lifestyles were more prevalent in those that did not participate. However, the percentage of participants in our sample that was overweight (5%) was lower than the national average (13–14%; Centraal Bureau voor de Statistiek, 2018), supporting this supposition. Possibly, the two interventions tried to increase physical activity in a sample that was already healthy and potentially more physically active, and did not target adolescents who could benefit the most from a physical activity intervention. This would explain why no differences were found between the social network intervention condition and the control condition in the short term.

Third, the mechanisms explaining how this social network intervention or mass media interventions could have influences behavior were not investigated. Mediation analyses of the three conditions on physical activity via the secondary outcomes were outside of the scope of this study. Mediation analyses would shed light on the role of the secondary outcomes of the intervention and could inform us whether the secondary outcomes are an underlying mechanism of social network interventions, or whether the secondary outcomes are due to the increase in physical activity. Future studies should systematically investigate (a) the manner(s) in which influence agents try to influence their classmates, and (b) the conditions in which classmates are influenced by the agents.

Last, the influence agents were free to create the content for the vlogs to their preference, which made the intervention message better tailored to the targeted group because the message comes from within the peer group. As a result, it is imaginable that the vlogs differed in the level of persuasiveness, which might explain why both intervention conditions did not outperform the control condition. But given that the two types of intervention conditions were exposed to the exact same vlogs, the differences between the two conditions in the exploratory analyses are promising for future research. When future studies are solely interested in using vlogs as intervention messages, it is advisable to first pretest the persuasiveness of the created vlogs before using them as stimulus material.

## Conclusion and Implications

In conclusion, our study did not provide evidence that exposing adolescents to vlogs made by influential classmates increased physical activity more than when adolescents were exposed to vlogs made by unfamiliar peers, or no vlogs at all. However, this social network intervention did have a positive effect on the perceived descriptive norm. Likewise, responses to the vlogs were more positive when the vloggers were influential classmates compared to unfamiliar peers. Potentially, this could be the added benefit of implementing a social network intervention approach over a mass media intervention.

Altogether, social network interventions may be a promising intervention type to promote physical activity in adolescents, but certain conditions must be satisfied before such interventions are effective. Future studies should investigate more closely when and why social network interventions work by investigating the training aspect of the intervention, the feasibility of online interventions for large-scale implementation, and the underlying mechanisms of social network interventions. Also, future studies should investigate the role of the perceived social norms and the added benefit of having influence agents within a social network intervention.

## Data Availability Statement

The datasets for this study can be found in the DANS archive: doi: https://doi.org/10.17026/dans-zz9-gn44.

## Ethics Statement

The studies involving human participants were reviewed and approved by Ethics Committee of the Radboud University (ECSW2014-100614-222). Written informed consent to participate in this study was provided by the participants’ legal guardian/next of kin. Written informed consent was obtained from the minor(s)’ legal guardian/next of kin for the publication of any potentially identifiable images or data included in this manuscript.

## Author Contributions

All authors contributed to the study design. LB, KB, CS, and TV performed the data collection. TV performed the data analyses and interpretation, drafted the manuscript. All co-authors provided critical revisions, approved the final version of the manuscript for submission. All authors agreed to be accountable for the content of the work.

## Conflict of Interest

The authors declare that the research was conducted in the absence of any commercial or financial relationships that could be construed as a potential conflict of interest.

## Supplementary Material

DATA SHEET S1 Supporting data and syntax.
